# The U-Curve of Happiness Revisited: Correlations and Differences in Life Satisfaction Over the Span of Life—An Empirical Evaluation Based on Data From 1,597 Individuals Aged 12–94 in Germany

**DOI:** 10.3389/fpsyg.2022.837638

**Published:** 2022-04-28

**Authors:** Christopher Karwetzky, Maren M. Michaelsen, Lena Werdecker, Tobias Esch

**Affiliations:** Department of Medicine, Faculty of Health, Institute for Integrative Health Care and Health Promotion, Witten/Herdecke University, Witten, Germany

**Keywords:** life satisfaction, momentary happiness, neurobiology, endogenous reward, U-shape, aging

## Abstract

**Background:**

Subjective wellbeing (SWB) is a research topic of growing interest for different disciplines. Based on a cross-sectional survey with 1,597 participants aged 12–94, this study investigated life satisfaction and momentary happiness, two important dimensions of SWB. We examined their relationship, shape, and correlates across individuals of different ages and interpreted the results in the light of a neurobiological model of motivation systems.

**Methods:**

Statistical analyses were performed using multiple linear regression. First, we examined how life satisfaction is associated with selected socio-demographic variables across four age groups. Second, we analyzed the association between life satisfaction and age, and lastly, we examined the extent to which happiness is a prerequisite for life satisfaction in each age group.

**Results:**

Our analyses show that life satisfaction correlates negatively with poor health and financial worries, and positively with partnership, grandchildren, and religiosity. However, the inverse relationship with poor health is stronger in younger than in older individuals, while the inverse association with financial worries is strongest in late midlife (50–69 years). We identified gender-specific differences concerning the relationship between life satisfaction and age, with males displaying a U-shape trend with its lowest point between the ages of 30 and 49, whereas females’ life satisfaction increases stepwise with age. Although momentary happiness correlates strongly with life satisfaction, this relationship decreases with age.

**Conclusion:**

The results suggest that individuals adjust or even grow beyond their perceptions of a “good life” over time. Neurobiological processes of adaptation and personal growth could play an important role in these developments.

## Introduction

This study contributes to the growing body of literature on subjective well-being research by assessing potential predictors of life satisfaction in and across four age groups, differentiating between momentary happiness and life satisfaction, and analyzing the relationship between these two concepts of SWB at different ages.

Understanding the determinants of a “good life” has been a subject of interest for more than 2,000 years. Aristotle, for example, formulated his philosophy of happiness in 330 BC, defining it as the highest goal of human existence and referring to it as *eudaimonia*—a reward for the life lived (Waterman, 1990). Since then, scientific research from various disciplines including psychology, behavioral sciences, economics, and neuroscience has contributed to a better understanding of subjective wellbeing (SWB), a “general term referring to the various types of subjective evaluations of one’s life, including both cognitive evaluations and affective feelings” (Diener et al., 2018, p. 3) and its many dimensions. From a large set of analyzed variables, strong relationships (Waldinger, 2015), sufficient income (Diener and Oishi, 2000), religiosity (Tay et al., 2014), and good self-reported health (Veenhoven, 2008; Sabatini, 2014) have consistently been found to be associated with SWB. For extensive literature reviews on these aspects, we refer to, e.g., Diener et al. (1999, 2018) and Eid and Larsen (2008).

A significant amount of attention has been invested in attempting to determine whether individuals can influence their SWB levels through intentional activities. While some authors (e.g., Lyubomirsky et al., 2005) estimate intentional activities to account for up to 40 percent of the variance in happiness, others estimate our potential to consciously influence SWB levels as being far lower (e.g., Brown and Rohrer, 2020). Authors such as Steel et al. (2008, 2019) or Pavot and Diener (2011) examined the interplay between personality and SWB and found that the Big Five personality traits—openness to experience, conscientiousness, extraversion, agreeableness, and neuroticism—explain much of the observed variation in SWB (about one-third in a review of Steel et al., 2008). As the personality itself is thought to contain a malleable component (Damian et al., 2019), its association with SWB could potentially change in individuals and this throughout the span of life. Despite many studies concerning the potential sources of SWB, the exact interplay between genetics, external life conditions, and influenceable activities is still a matter of debate.

Concerning the relationship between SWB and age, evidence from the past two decades and data from more than 140 countries predominantly indicates that SWB levels are U-shaped over the life span (Blanchflower and Oswald, 2004, 2008; Frijters and Beatton, 2012). Critics argue that the U-shape only exists under certain socio-economic conditions (Deaton, 2008), that it results from omitted cohort effects (Easterlin and Schaeffer, 1999), and that the results could be skewed by a “survival of the happy” (Segerstrom et al., 2016). Kassenboehmer and Haisken-DeNew (2012) critically remarked that panel studies (e.g., those based on the German Socio-Economic Panel) entail the risk of biases due to a lack of attention paid to heterogeneity, the type of questioning, and increasing interview experience (Kassenboehmer and Haisken-DeNew, 2012). However, several longitudinal studies (e.g., van Landeghem, 2012; Cheng et al., 2017), data from countries with very different incomes and social systems (Blanchflower, 2021), and studies that controlled for the biases mentioned above (Mroczek and Spiro, 2005; Easterlin, 2006) seem to confirm the hypothesis that the level of SWB is U-shaped over the individual span of life.

Although many studies have attempted to explain the determinants of SWB, many of them face substantial shortcomings. For example, they do not consider age effects, ignore dynamic processes of adaptation and personal growth, or conflate different definitions of SWB. In their literature review, Heidl et al. (2012) concluded that there are difficulties in establishing a uniform definition of happiness and that “due to difficult conceptual delimitations and ambiguities, some researchers use the meaning of the words (joy, happiness, contentment, well-being, satisfaction, bliss, or fun) in the same way.”

The present study distinguishes between two dimensions of SWB, namely, “life satisfaction” and “momentary happiness.” In this context, we understand life satisfaction as “people’s explicit and conscious evaluations of their lives,” and momentary happiness as “a person’s well-being derived from pleasure, and lowered by pain,” which is sometimes also referred to as hedonic wellbeing (Diener et al., 2018).

## Objectives

Inspired by the *neurobiological model of motivation systems* (Esch, 2017), we investigated correlates of life satisfaction across individuals of different ages and analyzed to what extent momentary happiness is a prerequisite for life satisfaction. In particular, we explored the following three research questions:

*Research Question 1 (RQ1)*: Does the relationship between selected socio-demographic variables and life satisfaction remain stable or change with age?

*Hypothesis 1*: The relationship between socio-demographic variables and life satisfaction is moderated by age.

*Research Question 2 (RQ2)*: How does life satisfaction relate to age?

*Hypothesis 2*: The relationship between life satisfaction and age is U-shaped.

*Research Question 3 (RQ3)*: Is the life satisfaction of older people less dependent on momentary happiness than the life satisfaction of younger people?

*Hypothesis 3*: The dependence between life satisfaction and momentary happiness decreases with increasing age.

The *neurobiological model of motivational systems* is a theory according to which life satisfaction and happiness are viewed as a result of dynamic biological processes of change, maturation, adaptation, and personal growth throughout life. According to this model, life satisfaction and momentary happiness are closely related, including enzymatic processing of relevant neurotransmitters, aligning with the biological course of life, but they do not necessarily presuppose each other. A detailed description of the model is provided in the interpretation section. Although we were guided by the neurobiological model in our hypothesis generation, the study results can also be interpreted independently of the model.

With this study, we aim to contribute to SWB and socio-psychological research in two different ways. Firstly, we aspire to facilitate a better understanding of SWB by analyzing potential predictors of life satisfaction in individuals of different ages and by evaluating the relationship between life satisfaction and momentary happiness. We believe that it is relevant to analyze cognitive reflections on life satisfaction in general (a trait component of SWB) and happiness just in this very moment (a state component of SWB) in one study, to analyze them separately and investigate their relationship in individuals of different ages. It is this distinction that enables us to explore how important momentary happiness (pleasure, peak moments, and positive moods) is for our life satisfaction in different ages.

Secondly, we strive to expand the discussion on the determinants of life satisfaction by integrating insights from modern neurobiological research into the science of SWB. To our knowledge, this is the first empirical study that uses a theoretical neurobiological model to explain age-related differences in life satisfaction and its predictors.

This article forms part of a larger study that aims to examine patterns and motives of a good and meaningful life in and across different age groups. The second study arm, which explores sources of meaning using a qualitative and quantitative approach, was recently published under the title “What matters most in life? A German cohort study on the sources of meaning and their neurobiological foundations in four age groups” (Karwetzky et al., 2021).

## Materials and Methods

### Participants

Our study uses data from a cross-sectional survey that was conducted from September 2017 to January 2018 for the purpose of this study.

Since the *neurobiological model of motivation systems* assumes that our perceptions of life satisfaction and happiness are influenced by biological adaptive processes throughout the whole span of life, our target population included people with a wide age range from 10 to 99 years. The study consisted only of individuals living in Germany. People with cognitive impairment (e.g., Alzheimer’s disease) were excluded from the study.

We primarily collected data online (*n* = 1,038) although participants could also complete the survey on paper by means of printed questionnaires (*n* = 559). Participants were recruited nationwide *via* social media, health magazines, radio, and TV broadcasts. Underage individuals were targeted in two schools in northern Germany (Wilhelmshaven), where teachers asked their students to fill out the printed questionnaires. Older adults were targeted in and outside four medical practices that were located across the country in four cities of different sizes (in addition to web-recruiting strategies). We provided comprehensive information on the study objectives and data protection to all participants and obtained the parents’ informed consent in cases where the study participants were underage (*n* = 40). The study obtained ethical approval from the ethics committee of the Witten/Herdecke University.

### Survey Instrument

The questionnaire consisted of two quantitative measures followed by the open question “What matters most to you in life?” which was assessed in the study’s second part (Karwetzky et al., 2021). The first quantitative item was “How happy are you at this moment?” followed by the question “How satisfied are you with your life in general?” The rationale for this formulation and order was to avoid a reflection on life or a longer period of time with regard to momentary happiness. Likewise, the question about general life satisfaction should encourage such reflections and not refer to the moment of response. The scale of both items ranged from zero (very unhappy/dissatisfied) to 100 (very happy/satisfied). We also collected numerous socio-demographic variables, namely, age, gender, occupational status, subjective health status (good/moderate/poor), partnership, religiosity/faith, financial status (regular financial worries/occasional financial worries/no financial worries), children, and grandchildren. Existing studies and literature reviews have reported relevant relationships between these variables and SWB, which is why they were included in the survey (e.g., Diener et al., 1999, 2018). However, the queried variables are only a selection of relevant explanatory variables, and significant relationships may exist between SWB and other variables not included in this study.

To validate our survey instrument, we pre-tested it with 15 individuals in 2017.

After completing the data collection, the first step of our analysis was to review the data for missing values and inconsistent content. The check did not reveal any irregularities.

### Statistical Analyses

All statistical analyses were performed using the program SPSS Statistics, Version 28. To investigate age-related differences in the variables associated with life satisfaction, the study population was divided into four age groups that reflect typical phases of life, such as youth and early adulthood, building a family and a professional career, late midlife, and retirement, and late life (see also, Karwetzky et al., 2021):

Age group 1: up to 29 years
Age group 2: 30 to 49 years
Age group 3: 50 to 69 years
Age group 4: 70 years or older

Methodologically, the three research questions were examined using the following statistical procedures:

*RQ1:* First, we assessed the relationships between life satisfaction and the queried socio-demographic variables by means of multiple linear regression analysis. To explore whether the observed associations are moderated by age, interactions of socio-demographic variables with the four age groups were added to the model. To keep the model concise, interactions were only included for variables with significant main effects in the first model.

*RQ 2:* To analyze the relationship between life satisfaction and age, we added age squared to the first regression model from RQ 1 as the inclusion of a quadratic term into an otherwise standard regression is a common statistical procedure to identify U-shaped relationships (Lind and Mehlum, 2010).

*RQ 3:* To examine the relationship between life satisfaction and happiness in and across four age groups, we calculated a regression using life satisfaction as the dependent and happiness, socio-demographic variables, and the interactions between happiness and the four age groups as independent variables.

**Figure 1:**
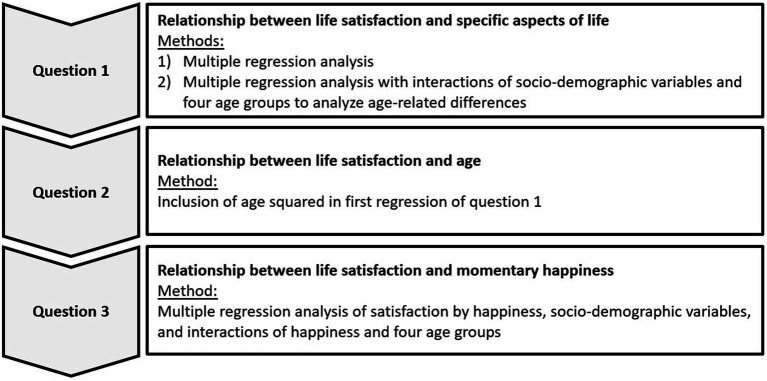
Overview of the research approach.

Except for age, all independent variables had a categorical measurement level and were therefore dummy-coded. In alignment with authors such as Ng (1997) and Ferrer-i-Carbonell (2002), we assumed the life satisfaction scales’ cardinality. Figure 1 summarizes the statistical procedures that were selected to address the research questions.

Intercorrelations among the independent variables were calculated to rule out excessive multicollinearity, as shown in the results in Section “Correlations Between the Variables.”

The reported regression coefficients are unstandardized on a scale from 0 to 100. To assess the effect size of age squared (RQ 2), we also reported standardized coefficients.

### Descriptive Statistics

Table 1 shows the distribution of our study population and the socio-demographic variables.

**Table 1:** Socio-demographic variables of the study population.

	Number of participants (%)
Socio-demographic variables	1,597 (100)
Age group 1	371 (23)
Age group 2	558 (35)
Age group 3	506 (32)
Age group 4	162 (10)
Distribution of variables (reference categories shown in italics)	
Female	1,004 (63)
*Male*	*593 (37)*
Partnership	1,121 (70)
*No partnership*	*436 (27)*
n.a.	40 (3)
Children	891 (56)
*No children*	*706 (44)*
Grandchildren	273 (17)
*No grandchildren*	*1,324 (83)*
Poor health	117 (7)
Moderate health	402 (25)
*Good health*	*1,078 (68)*
Religiosity/Faith	647 (41)
*Not religious/not adhering to any belief*	*950 (59)*
Working/studying	1,217 (76)
*Not working/not studying*	*380 (24)*
Regular financial worries	147 (9)
Occasional financial worries	539 (33)
*No financial worries*	*911 (56)*
n.a.	40 (2)

n.a., no answer (underage study participants were not asked about a partnership and financial worries).

**Figure 2:**
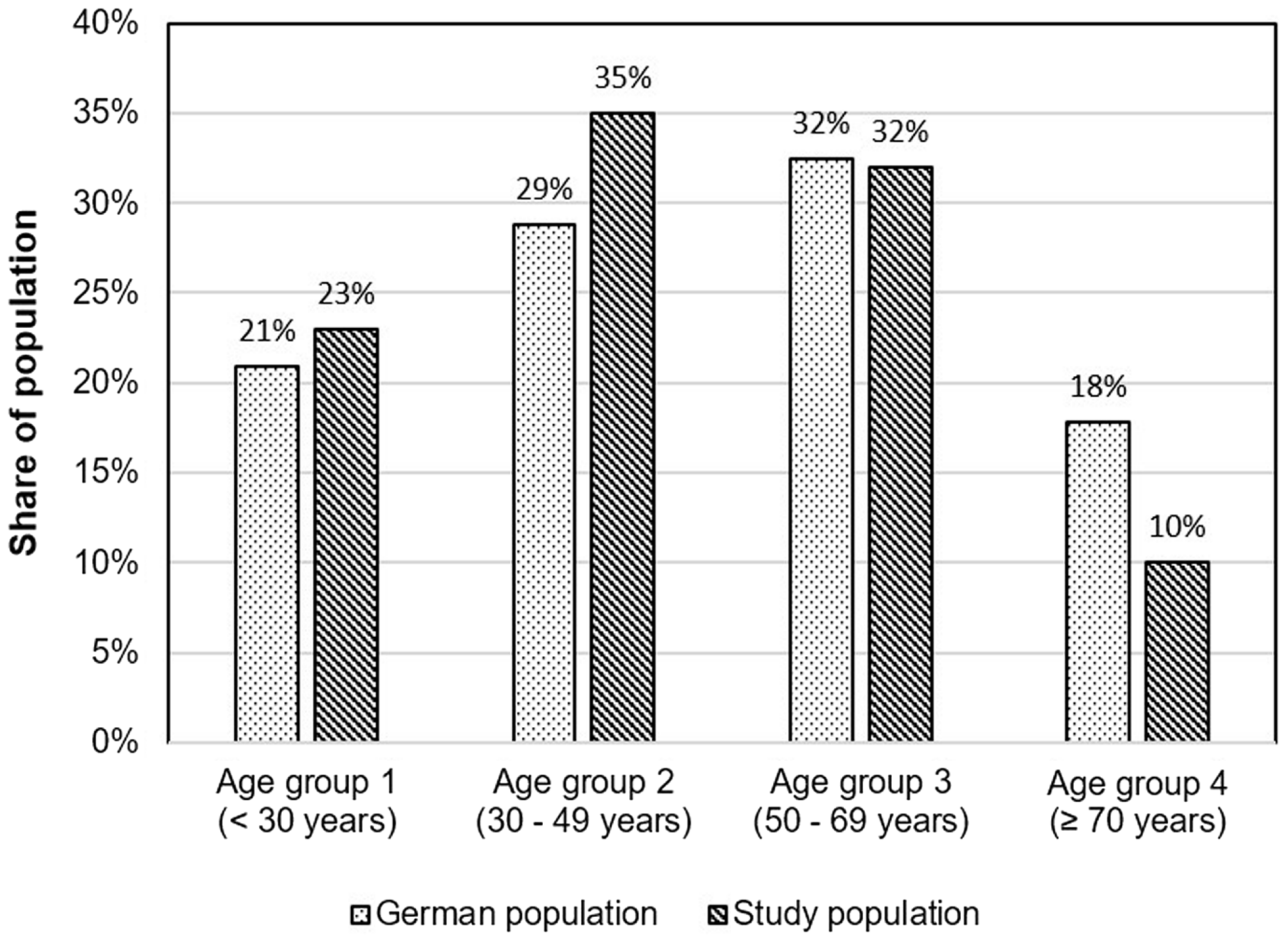
Comparison of the age distribution of the study participants with that of the overall German population.

Although the sample largely represents the demographic distribution in Germany, the participants in Age group 2 were slightly over-represented and the participants in Age group 4 were slightly under-represented (Figure 2; German Federal Statistical Office, 2018). Overall, more females (1,004) than males (593) participated in the survey.

Table 2 shows the descriptive statistics for the three metric variables life satisfaction, momentary happiness, and age.

**Table 2:** Statistics for the three metric variables life satisfaction, momentary happiness, and age.

Variable	Min.	Max.	Mean	Standard deviation
Life satisfaction	0	100	75.33	21.11
Momentary happiness	0	100	68.32	22.31
Age	12	94	40.34	18.08

The mean value of life satisfaction was 75.33, which was 7 points higher than the measure for momentary happiness. Simultaneously, the standard deviations were high at 21.11 and 22.31 points. The values collected were comparable to the results of other representative surveys, such as those of the German Socio-Economic Panel which found an average life satisfaction of approximately 7.5 on a scale of 0–10 for 2015 (SOEP, 2018).

## Results

### Correlations Between the Variables

Table 3 shows the Pearson correlation coefficients between all independent and dependent variables. Even though some substantial correlations existed, there was no excessive collinearity, which would negatively influence the following regression models’ interpretation. The highest correlations were found between life satisfaction and happiness (*r* = 0.66, *p* < 0.001), age and grandchildren (*r* = 0.58, *p* < 0.001), and age and children (*r* = 0.57, *p* < 0.001).

**Table 3:** Correlations between dependent and independent variables.

	Gender	Partnership	Children	Grand-children	Religiosity/Faith	Working/Studying	Subjective health	Financial worries	Age	Happiness	Life satisfaction
Gender	1										
Partnership	−0.02	1									
Children	−0.03	0.28	1								
Grandchildren	−0.12	0.07	0.38	1							
Religiosity/Faith	0.04	0.03	0.15	0.12	1						
Working/Studying	0.16	0.01	−0.22	−0.46	−0.11	1					
Subjective health	−0.06	−0.03	0.08	0.15	0.02	−0.27	1				
Financial worries	0.08	−0.02	<0.01	−0.07	−0.05	<0.01	0.17	1			
Age	−0.13	0.16	0.57	0.58	0.20	−0.51	0.20	−0.10	1		
Happiness	−0.03	0.12	0.05	0.08	0.06	−0.01	−0.35	−0.21	0.04	1	
Life satisfaction	−0.02	0.16	0.12	0.13	0.09	−0.02	−0.36	−0.38	0.15	0.66	1

### Relationship Between Life Satisfaction and Specific Aspects of Life (RQ 1)

The regression from Table 4 investigates the relationship between life satisfaction and the queried socio-demographic variables. Significant associations were found for the variables poor subjective health (*B* = −23.638, 95% CI: −27.199 to −20.077), regular financial worries (*B* = −22.815, 95% CI: −26.021 to −19.609), partnership (*B* = 5.168, 95% CI: 3.166–7.169), grandchildren (*B* = 4.173, 95% CI: 1.184–7.162), religiosity (*B* = 2.070, 95% CI: 0.247–3.893), and age (*B* = 0.102, 95% CI: 0.030–0.175). The variables female, children, and working/studying had no additional effect on life satisfaction. More detailed results including the exact standard errors and *p*-values are provided in Tables 4 and 5.

**Table 4:** Regression of life satisfaction by socio-demographic variables.

Independent variables	*B*	SE	*p*	95% CI	
				Lower bound	Upper bound
Age	0.102	0.037	0.006^**^	0.030	0.175
Female	0.078	0.943	0.934	−1.771	1.927
Partnership	5.168	1.020	<0.001^***^	3.166	7.169
Children	1.042	1.145	0.363	−1.203	3.287
Grandchildren	4.173	1.524	0.006^**^	1.184	7.162
Religiosity/Faith	2.070	0.929	0.026^**^	0.247	3.893
Working/Studying	−1.365	1.298	0.293	−3.910	1.181
Poor health	−23.638	1.816	<0.001^***^	−27.199	−20.077
Moderate health	−1.545	1.073	<0.001^***^	−12.649	−8.441
Good health	0				
Regular financial worries	−22.815	1.635	<0.001^***^	−26.021	−19.609
Occasional financial worries	−6.664	0.980	<0.001^***^	−8.587	−4.742
No financial worries	0				
(Constant)	74.666	2.234	<0.001^***^	7.284	79.048
Adj. *R*^2^	0.289				
*N*	1,597				
*F*	57.877				

SE, standard error; CI, confidence interval.

** *p* < 0.05;

*** *p* < 0.001.

**Table 5:** Regression of satisfaction by demographic variables and interactions of age groups with demographic variables.

Independent variables	*B*	SE	*p*	95% CI	
				Lower bound	Upper bound
Female	0.417	0.959	0.664	−1.464	2.299
Partnership^1^	3.859	1.891	0.041^**^	0.149	7.569
Children	2.126	1.201	0.077	−0.229	4.481
Religiosity/Faith^1^	1.691	2.065	0.413	−2.360	5.741
Working/Studying	−0.715	1.366	0.601	−3.395	1.965
Poor health^1^	−28.102	4.045	<0.001^***^	−36.036	−20.168
Moderate health^1^	−15.383	2.296	<0.001^***^	−19.886	−10.880
Good health	0				
Regular financial worries^1^	−16.651	3.231	<0.001^***^	−22.989	−10.312
Occasional financial worries^1^	−6.825	2.030	<0.001^***^	−10.807	−2.842
No financial worries	0				
Age group 1	0				
Age group 2	−0.911	2.587	0.725	−5.985	4.163
Age group 3	−3.280	2.821	0.245	−8.813	2.254
Age group 4	5.319	4.708	0.259	−3.917	14.554
Interactions					
Partnership × Age group 2	1.548	2.642	0.558	−3.635	6.731
Partnership × Age group 3	3.349	2.652	0.207	−1.853	8.551
Partnership × Age group 4	−1.378	3.822	0.719	−8.874	6.118
Grandchildren × Age group 2	13.283	8.948	0.138	−4.269	30.835
Grandchildren × Age group 3	2.808	1.785	0.116	−0.692	6.309
Grandchildren × Age group 4	4.354	3.254	0.181	−2.029	10.737
Religious × Age group 2	0.256	2.583	0.921	−4.811	5.322
Religious × Age group 3	0.788	2.599	0.762	−4.310	5.886
Religious × Age group 4	−3.770	3.581	0.293	−10.794	3.253
Poor health × Age group 2	0.710	5.526	0.898	−10.130	11.549
Poor health × Age group 3	5.729	4.947	0.247	−3.975	15.433
Poor health × Age group 4	14.644	5.915	0.013^**^	3.042	26.245
Moderate health × Age group 2	2.797	3.011	0.353	−3.108	8.703
Moderate health × Age group 3	6.906	2.914	0.018^**^	1.190	12.622
Moderate health × Age group 4	13.417	3.848	<0.001^***^	5.869	20.965
Regular financial worries × Age group 2	−6.291	4.157	0.130	−14.445	1.863
Regular financial worries × Age group 3	−9.298	4.448	0.037^**^	−18.023	−0.573
Regular financial worries × Age group 4	0.303	8.805	0.973	−16.968	17.573
Occasional fin. worries × Age group 2	−1.396	2.613	0.593	−6.522	3.730
Occasional fin. worries × Age group 3	3.110	2.648	0.240	−2.085	8.304
Occasional fin. worries × Age group 4	−2.224	4.097	0.587	−10.259	5.811
(Constant)	78.530	2.181	<0.001^***^	74.251	82.809
Adj. *R*^2^	0.297				
*N*	1,597				
*F*	21.445				

SE, standard error; CI, confidence Interval.

1 Variables with significant main effects in regression model without interactions (Table 4).

** *p* < 0.05;

*** *p* < 0.001.

Following this first analysis, we investigated whether the identified coefficients differ in relation to age. Table 5 shows the results of a regression that used life satisfaction as the dependent variable and the socio-demographic variables and their interactions with age as independent variables. For interpretation, the interaction’s coefficient must be added to the coefficients of the respective reference group and a non-significant coefficient means that no difference exists between an age group and the reference Age group 1 concerning this specific variable.

The analysis revealed a positive coefficient for the interaction of moderate health status with Age group 3 (*B* = 6.906, 95% CI: 1.190–12.622) and Age group 4 (*B* = 13.417, 95% CI: 5.869–20.965), thereby indicating that the negative association between moderate health and satisfaction is significantly smaller in older than in younger individuals. Furthermore, we found a positive coefficient for the interaction between Age group 4 and poor health (*B* = 14.644, 95% CI: 3.042–26.245), suggesting that even subjectively severe health problems are correlated less strongly with life satisfaction in older than in younger people. Concerning regular financial worries, we found a negative coefficient for the interaction with Age group 3 (*B* = −9.298, 95% CI: −18.023 to 0.573), which indicates that the negative relationship between financial worries and life satisfaction is significantly more pronounced in this age group.

### Relationship Between Life Satisfaction and Age (RQ 2)

According to the results presented in Table 4, life satisfaction is positively associated with age at *B* = 0.102 (95% CI: 0.030–0.175), thereby suggesting that life satisfaction increases with age. To analyze this relationship in more detail, we added the age squared values to the first regression model. In this supplemented model, the coefficient of age squared was *B* = 0.006 (95% CI: 0.003–0.009), indicating the presence of a U-shaped relationship. Supplementary Table 1 shows the complete results of this analysis.

**Figure 3:**
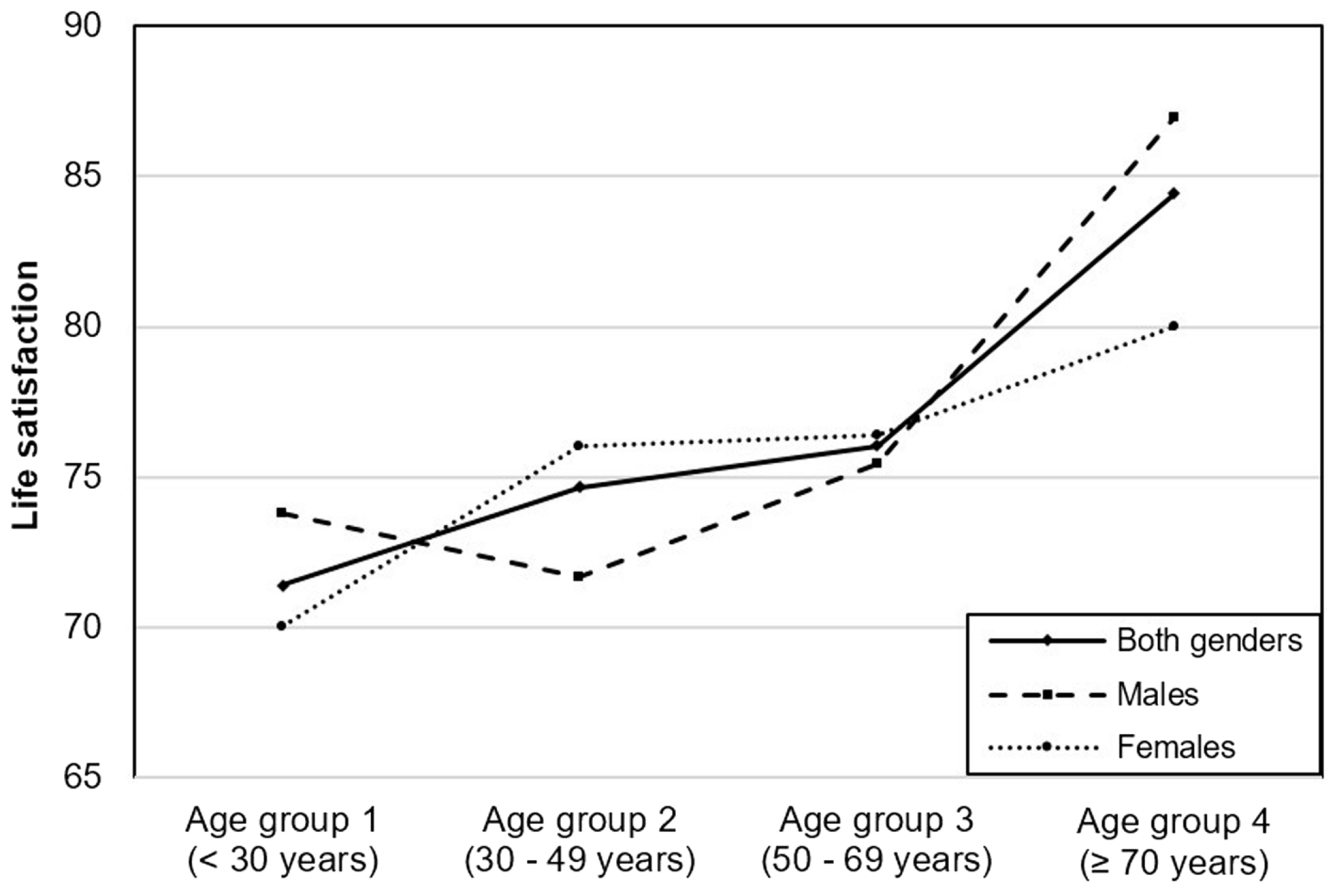
Average levels of life satisfaction in different age groups.

Figure 3 illustrates the mean satisfaction values across Age groups 1–4 for the entire study population, including the values for both males and females. While men showed a pronounced U-shaped relationship for life satisfaction, with the lowest point in Age group 2, a two-step upward trend could be observed in the female study participants.

### Relationship Between Life Satisfaction and Momentary Happiness (RQ 3)

Following the analysis of life satisfaction and age, we investigated the relationship between life satisfaction and momentary happiness, moderated by age. This fourth regression model aimed to determine how satisfaction and happiness relate to each other and to analyze if their relationship differs in participants of different age groups.

The regression in Table 6 used satisfaction as the dependent variable and happiness, the demographic variables, and the interactions of happiness with Age groups 1–4 as independent variables. The table shows a strong positive association between life satisfaction an happiness in Age group 1 (*B* = 0.578, 95% CI: 0.509–0.646). In contrast, the interactions of happiness and Age groups 3 and 4 had negative coefficients (*B* = −0.093, respectively *B* = −0.104), indicating that the association between happiness and life satisfaction is significantly weaker in these age groups than in Age group 1. Supplementary Figure 1 illustrates the relationship between life satisfaction and happiness in the four age groups, showing that for any given level of momentary happiness, older individuals reach a higher level of life satisfaction than younger individuals.

**Table 6:** Linear regression model for the dependent variable life satisfaction and happiness, socio-demographic variables, and interactions of happiness with different age groups as independent variables.

Independent variables	*B*	SE	*p*	95% CI	
				Lower bound	Upper bound
Happiness	0.578	0.035	<0.001^***^	0.509	0.646
Female	0.769	0.771	0.319	−0.744	2.282
Partnership	3.142	0.841	<0.001^***^	1.493	4.792
Children	1.747	0.966	0.071^*^	−0.149	3.642
Grandchildren	1.343	1.279	0.294	−1.166	3.852
Religiosity/Faith	0.882	0.757	0.244	−0.604	2.367
Working/Studying	1.174	1.098	0.285	−0.980	3.329
Poor health	−9.634	1.561	<0.001^***^	−12.697	−6.571
Moderate health	−4.993	0.898	<0.001^***^	−6.754	−3.231
Good health	0				
Regular financial worries	−16.449	1.351	<0.001^***^	−19.098	−13.800
Occasional financial worries	−5.171	0.802	<0.001^***^	−6.743	−3.598
No financial worries	0				
Age Group 2	4.820	3.195	0.132	−1.447	11.088
Age Group 3	8.787	3.226	0.007^**^	2.460	15.115
Age Group 4	14.161	4.772	0.003^**^	4.801	23.520
Interactions					
Happiness × Age Group 2	−0.073	0.044	0.100	−0.160	0.014
Happiness × Age Group 3	−0.093	0.045	0.036^**^	−0.181	−0.006
Happiness × Age Group 4	−0.104	0.061	0.089^*^	−0.224	0.016
(Constant)	34.511	2.834	<0.001^***^	28.952	4.071
Adj. *R*^2^	0.531				
*N*	1,597				
*F*	107.100				

SE, standard error; CI, confidence Interval.

* *p* < 0.1;

** *p* < 0.05;

*** *p* < 0.001.

**Figure 4:**
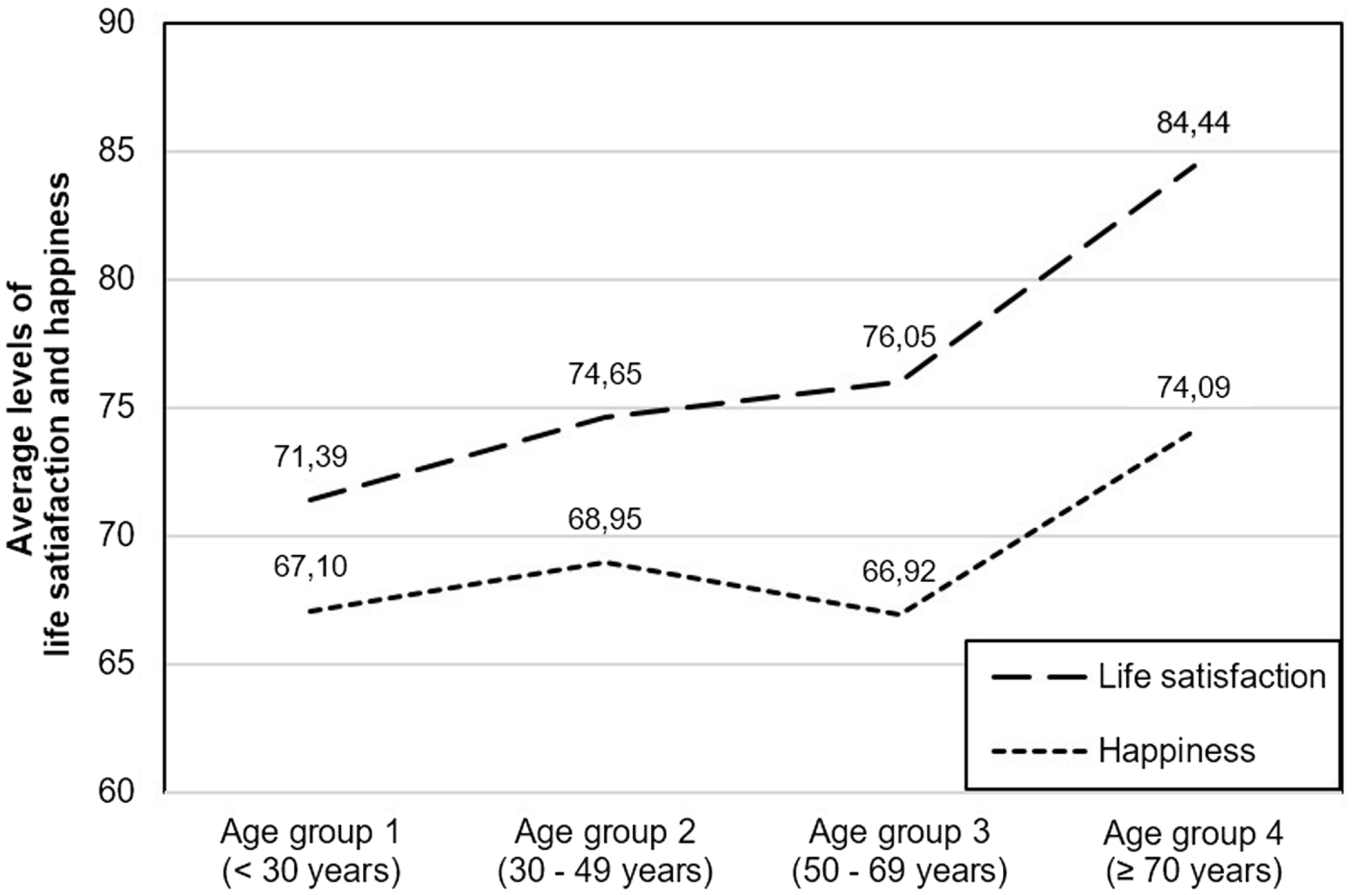
Average levels of life satisfaction and momentary happiness in different age groups.

Figure 4 illustrates the average life satisfaction and happiness values across the four age groups. These results clearly show how the difference between both concepts of SWB increases with the participants’ age.

## Discussion

### Interpretation of the Main Results

This study’s primary objective was to explore whether selected socio-demographic aspects are associated with life satisfaction and if so, to analyze whether such associations are stable across individuals of different age groups. Among all age groups, good health, the absence of financial worries, and religiosity showed robust correlations with life satisfaction. In addition, we found significant associations with partnership and grandchildren, which highlights the importance of relationships for our wellbeing. Interestingly, however, having children was not associated with life satisfaction, which is a finding that is consistent with previous research claiming that the positive and negative effects of having children on subjective wellbeing balance each other out (Hansen, 2012; Deaton and Stone, 2014; Ugur, 2020). However, this aspect is still a matter of debate since generativity, which requires having generations to follow, has been shown to be positively correlated with SWB (Navarro-Prados et al., 2018; Moieni et al., 2020). From the data, we did not obtain significant results concerning the relationship between occupational status and life satisfaction. Overall, the socio-demographic variables included in this study explain about 29 percent of the observed variance in life satisfaction, which is substantial. In comparison with the study’s qualitative part (Karwetzky et al., 2021), both analyses highlight the importance of health, partnership, and generativity for our SWB.

Taking age into account in the analysis, we obtained some notable results. Having a partnership and being religious/faithful were associated with increased life satisfaction regardless of age, whereas regular financial worries were most negatively associated with life satisfaction in Age group 3 (50–69 years). Furthermore, we found that the negative association between life satisfaction and moderate or even poor health status was significantly weaker among older than among younger individuals. One possible explanation is that older people might consider having some health problems as being an inevitable part of the aging process, and thus either perceive them as being less impairing or simply learn to deal with them as time passes.

Concerning the second objective of this study, namely, the analysis of the relationship between life satisfaction and age, we identified a U-shaped trend with the lowest point found in Age group 2 (30–49 years) for male participants. For females, we observed two stepwise increases in life satisfaction; the first increase in Age group 2 (30–49 years) and a second in Age group 4 (70 years or older).

Regarding the third study objective, we observed that life satisfaction and happiness are closely related, with a Beta of 0.578 in Age group 1. In Age group 3 (Beta = 0.484) and Age group 4 (Beta = 0.473), their relationship was significantly weaker, which suggests that momentary happiness is less of a prerequisite for life satisfaction in older than in younger people.

Overall, the results of this study show that satisfaction levels and correlations between life satisfaction, momentary happiness, and socio-demographic variables vary with age, confirming hypotheses H1 and H3. H2 could be confirmed for male participants.

### Life Satisfaction and Happiness in the Context of the Neurobiological Model of Motivation Systems

As indicated in the introductory sections of this paper, the *neurobiological model of motivation systems* (Esch, 2017; Michaelsen and Esch, 2021) could help to explain the patterns observed in our analysis. According to this model, life satisfaction and happiness result from lifelong maturing processes driven by constant endogenous rewards and motivation cycles. Neurophysiologic processes assist our maturation by chemically and biologically rewarding the “right” practices and associated experiences, i.e., trajectories of a “good” or “fulfilling” life.

The model distinguishes three levels of motivation (A–C), which are united by the aim of advancing personal and biological growth throughout the various phases of life. After starting life with extensive freedom, adaptation potential, and a brain that remains inadequately prepared for the concrete challenges of life, we need to learn and adapt our inner structure to the outer world (neuroplasticity). During this first phase, we are biologically flexible. We want to evolve, explore the unknown, and constantly absorb the various impressions that life has to offer (*type A motivation*: the “wanting system”). With progressive adaptation and maturation, our stress physiology is activated, and we strive to defend what has been gained (and has been cast into our structure) rather than conquer new territories. We long for security and protection and want to avoid harm and anxiety (*type B motivation*: the “threat avoidance system”). If we succeed in adapting to our environment and in maturing internally, the development of deep and persistent satisfaction is possible (*type C motivation*: the “non-wanting” or “quiescence system”).

Supplementary Figure 2 illustrates the model and specifies the neuronal structures and transmitters involved in each phase. Regarding the neuroendocrinology of motivation and behavior, we would like to refer to an insightful review by McCall and Singer (2012) and the synopsis of involved neurotransmitters presented in the textbook The Neurobiology of Happiness by Esch (2017).

As part of the neurobiological model, Esch advocates making a distinction between momentary happiness and life satisfaction. While happiness is characterized by intense, pleasurable, and euphoric but fleeting moments, satisfaction is more profound, persistent, and subtle, and is characterized, among others, by feelings of acceptance, affiliation, arrival, and quiescence (Esch, 2017). The model suggests that life satisfaction includes an affective and a cognitive component. Supported by the endogenous reward system, we *feel* life as a whole as being satisfying, contenting, and/or gratifying. This affective experience represents a primary neurobiological function. The cognitive evaluation or appraisal, however, takes place second, is associative, and facilitated by the secondary and associative brain areas. While the questionnaire of this study concentrated on the cognitive component of life satisfaction (“How satisfied are you with your life *in general*?”) other studies have successfully addressed the affective component. For example, the Heidelberg Centenarian Study was able to show that people can experience a positive vision of life even with substantial cognitive limitations (Jopp and Rott, 2006).

Supported by neurobiological adaptation processes, age and progressive maturation lead to greater differentiation between life satisfaction and momentary happiness, as shown by their decreasing relationship in Research Question 3. Specifically, the regression results had shown that the relationship between the two dimensions is significantly lower in Age groups 3 and 4 than in Age groups 1 and 2. According to the model, the stress experienced in phases A and B of life is physiologically a prerequisite for deep and prolonged satisfaction later in life, i.e., phase C.

The increase in life satisfaction observed in the second half of life may seem counterintuitive, as this period is often characterized by increasing physical complaints and the onset of chronic diseases. Consequently, social researchers and gerontologists have called this phenomenon a “satisfaction paradox” (e.g., Schilling, 2006; Ferring, 2015). The neurobiological model, however, explains why the increase observed might be less paradoxical than commonly assumed. As we get older, “our needs and desires are getting satisfied more cost-effectively and efficiently with the help of a brain that is becoming ever better adapted” to our social environment (Esch, 2017, p. 134).

Argyle (2001) has a different theory concerning this phenomenon and assumes that as life progresses we adapt and manage our expectations, accept life’s circumstances, and reduce the *goal-achievement gap* that causes unhappiness or dissatisfaction. However, at best, Argyle’s hypothesis only explains why a decline in life satisfaction may be prevented despite increasing physical limitations, whereas the neurobiological model, in contrast, could also explain the observed increase in satisfaction above baseline levels. As analyses based on the Socio-Economic Panel have shown, life satisfaction begins to decline again a few years before a death caused by prolonged illness (Gerstorf and Wagner, 2010). Before this stage, however, major or severe depression is less common among older than among younger individuals (Thomas et al., 2016; von Hirschhausen and Esch, 2018), whereas life satisfaction is commonly high.

Like some other authors (e.g., Frijters and Beatton, 2012; Blanchflower, 2021), we observed a decrease in life satisfaction from early adulthood until midlife for some, especially male study participants. According to the neurobiological model, early adulthood is a phase in which most people experience the seriousness and challenges of life (i.e., stress). At this stage of our lives, we need to assume responsibility for ourselves, our children, and sometimes also our parents. We usually work more and start feeling the burden of financial pressures. Consequently, the two stress axes originating in our brain activate the body’s stress physiology (“allostatic stress response”); we are alert and ready to fight the dangers and difficulties of life (Esch and Stefano, 2004; Stefano et al., 2005). Despite being biologically necessary, the consequences of an uncontrolled stress response, or simply too much stress, can be a halt or even decrease in life satisfaction until the brain adapts to the new situation, learns, and allows life satisfaction to increase again.

### Limitations

When interpreting the results, the limitations of this study should be considered, the first of which is the cross-sectional study design used. As we surveyed participants only once, we are unable to analyze developments in their individual emotional lives over time. Furthermore, a frequent criticism raised against the use of cross-sectional surveys is the risk of hidden cohort effects, as individuals of a particular generation might respond similarly due to shared beliefs and life experiences (Parry and Urwin, 2017). However, the robustness of our results appears to be supported by the findings of various longitudinal studies that have reported increasing or U-shaped life satisfaction values over age, even in different countries and cohorts, with persons who faced very different historical and cultural circumstances and developments (Blanchflower and Oswald, 2008; van Landeghem, 2012; Sutin et al., 2013; Clark and Oswald, 2016; Cheng et al., 2017). Our findings are further supported by Carstensen et al. (2011), who in their 10-year longitudinal survey found that aging goes hand-in-hand with a more balanced emotional life and an increase in life satisfaction. With regard to the findings of Kassenboehmer and Haisken-DeNew (2012), who showed life satisfaction to be more stable over the life span after controlling the GSOEP data for interviewer effects, we point out that such effects played no role in our study due to the data collection method that was utilized. Nevertheless, it would be desirable to corroborate the study results obtained with longitudinally collected data and thus explore changes over the life course rather than differences between individuals.

A second limitation concerns the possibility of a selection bias. Since the study design primarily solicited voluntary participation *via* the internet, little influence could be exerted on the composition of our study population. Although online surveys are a standard procedure in research, they risk excluding individuals who do not have an internet connection or feel less comfortable with online questionnaires. Despite the fact that we complemented our online recruiting strategy with printed questionnaires in public places, such as schools and medical practices, follow-up studies should try to overcome this limitation, for example, through a more stratified sample based on data from civil records. With regard to the place of residence, we can rule out the possibility that its size would have biased the regression results.

Finally, from the results, we could not determine whether the independent endogenous variables (e.g., health and religiosity) cause life satisfaction or are caused by it. Many studies and articles do not make clear distinctions between endogenous and exogenous variables and yet draw explicit causal inferences (e.g., “What causes life satisfaction?” and “What makes us happy?”). However, due to the complexity of causality in this context, we recommend more caution in drawing conclusions from these aspects at present.

### Recommendations for Further Research

This study provides a starting point that enables future research to consider the changing relationship between life satisfaction, momentary happiness, physical health, and financial aspects with increasing age. One recommendation for future research is to explore why people go through the developmental steps and changes highlighted in this study at different rates. Why, for example, do some people seem to mature quickly during or after severe illnesses or other traumatic events (“posttraumatic growth”), while others struggle for years? Future research in this area can build on existing work on stress-driven or posttraumatic growth, such as that by Tedeschi and Calhoun (1996, 2004), Tomich and Helgeson (2004), and Carroll (2014).

Due to the large age range (12–94 years), the present study made a rather coarse distinction between four age groups. We therefore suggest that future studies look more closely at specific phases of life, for example, by distinguishing between adolescents and young adults or by examining older individuals in more detail.

Third, we suggest replicating this study design in other countries and cultures to account for regional differences and better understand them. In particular, we recommend empirically testing whether a weakening relationship between satisfaction and poor subjective health also exists in countries with less developed social security systems.

## Conclusion

This article forms the second part of a study on the patterns and motives life satisfaction and happiness, two important dimensions of SWB. Based on data from 1,597 study participants, we assessed whether selected aspects of life are associated with life satisfaction and whether such associations are stable across four different age groups. We found that good subjective health, partnership, religiosity, and the absence of financial worries were strongly related to life satisfaction. However, the negative association between compromised health and life satisfaction was significantly weaker in older individuals, while financial worries correlated most strongly with (low) life satisfaction between 50 and 69 years of age. Furthermore, we observed higher satisfaction levels and a weakening relationship between life satisfaction and momentary happiness among older individuals. Interpreting the results in the light of the *neurobiological model of motivation systems*, we argue that endogenous reward and motivation could play an important role in the age-related differences observed.

The second article of this study (Karwetzky et al., 2021) identified *sources of meaning* in and across the same four age groups and confirmed that perceptions of a good and meaningful life differ significantly depending on age.

## Data Availability Statement

The datasets presented in this article are not readily available because the Witten/Herdecke University’s Ethics Committee did not allow the data to be made publicly available. Requests to access the datasets should be directed to TE (tobias.esch@uni-wh.de).

## Ethics Statement

The studies involving human participants were reviewed and approved by Witten/Herdecke University’s Ethics Committee. Written informed consent to participate in this study was provided by the participants’ legal guardian/next of kin.

## Author Contributions

CK: conceptualization, investigation, formal analysis, and writing. MM and LW: conceptualization, methodology, review, and editing. TE: conceptualization, project administration, supervision, review, and editing. All authors contributed to the article and approved the submitted version.

## Conflict of Interest

The authors declare that the research was conducted in the absence of any commercial or financial relationships that could be construed as a potential conflict of interest.

## Publisher’s Note

All claims expressed in this article are solely those of the authors and do not necessarily represent those of their affiliated organizations, or those of the publisher, the editors and the reviewers. Any product that may be evaluated in this article, or claim that may be made by its manufacturer, is not guaranteed or endorsed by the publisher.
