# Supplementary material for: The U-Curve of Happiness Revisited: Correlations and Differences in Life Satisfaction Over the Span of Life—An Empirical Evaluation Based on Data From 1,597 Individuals Aged 12–94 in Germany
Source: Front Psychol. 2022 Apr 28;13:837638. doi: 10.3389/fpsyg.2022.837638 (PMC9096900; doi:10.3389/fpsyg.2022.837638)
Supplement: Supplementary file 1 [file Data_Sheet_1.pdf]

Supplementary Material

Supplementary Figure 1. Regression lines for life satisfaction and happiness in different age groups.

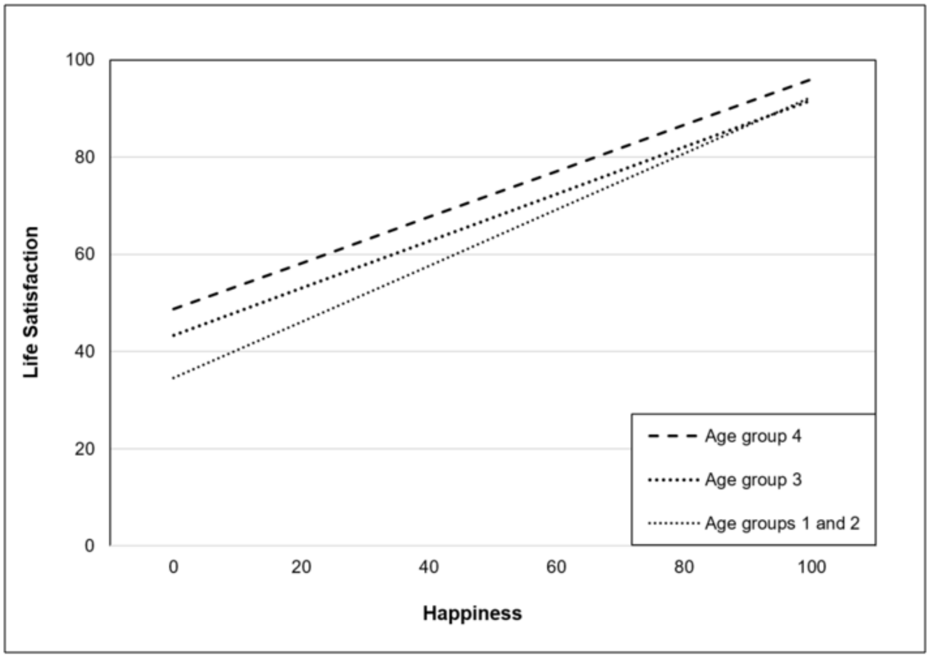

Interpretation:

For any given level of momentary happiness, older individuals reach a higher level of life satisfaction than younger individuals. No significant difference was found between Age groups 1 and 2.

Supplementary Figure 2: Neurobiological model of motivation systems

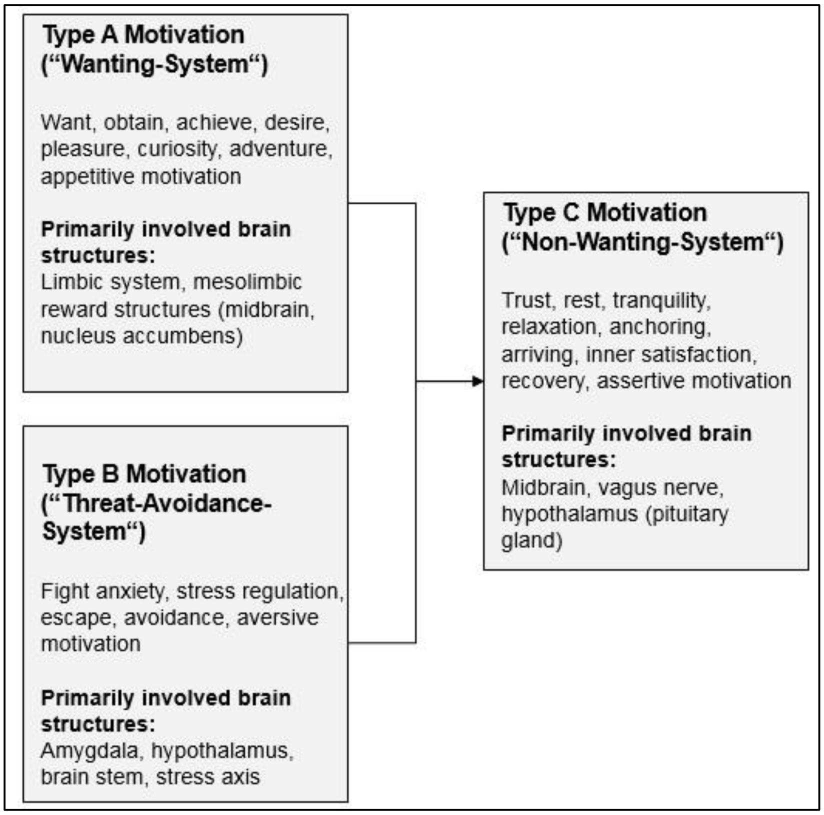

Supplementary Table 1. Regression for life satisfaction and different socio-demographic variables, including age squared.

	Unstandardized coefficients		Standardized coefficients			95 % CI	
**Independent variables**	**B**	**SE**	**Beta**	***P***		**Lower bound**	**Upper bound**
Age	–.458	.138	–.397	.001	**	–.729	–.187
**Age squared**	**.006**	**.001**	**.524**	**<.001**	*****	**.003**	**.009**
Female	.615	.946	.014	.516		–1.242	2.471
Partnership	5.889	1.029	.128	<.001	***	3.870	7.908
Children	2.341	1.180	.055	.047	**	.027	4.655
Grandchildren	2.165	1.589	.039	.173		–.952	5.282
Religiosity/Faith	1.737	.928	.040	.061		–.083	3.557
Working/Studying	.633	1.375	.013	.645		–2.065	3.331
Poor health	–23.766	1.806	–.294	<.001	***	–27.309	–20.223
Moderate health	–1.869	1.070	–.224	<.001	***	–12.968	–8.771
Good health	<.001						
Regular financial worries	–22.158	1.633	–.304	<.001	***	–25.362	–18.954
Occasional financial worries	–6.389	.977	–.143	<.001	***	–8.306	–4.473
No financial worries	<.001		–.397				
(constant)	82.585	2.912	.524	<.001	***	76.875	88.296
Adj. R^2	.296						
N	1,597						
F	56.944						

** $p < .05$; *** $p < .001$
SE: Standard error
CI: Confidence intervals

Interpretation:

The positive coefficient of Age squared indicates a highly significant, U-shaped relationship between life satisfaction and age. The effect is of medium size with a beta of .524.
